# The neutrophil-lymphocyte ratio has a role in predicting the effectiveness of nivolumab in Japanese patients with metastatic renal cell carcinoma: a multi-institutional retrospective study

**DOI:** 10.1186/s12894-020-00679-2

**Published:** 2020-07-25

**Authors:** Naotaka Nishiyama, Megumi hirobe, Takuya Kikushima, Masahiro Matsuki, Atsushi Takahashi, Masahiro Yanase, Keisuke Ichimatsu, Masayuki Egawa, Norihiro Hayashi, Takahito Negishi, Naoya Masumori, Hiroshi Kitamura

**Affiliations:** 1 Department of Urology, Faculty of Medicine, University of Toyama, 2630 Sugitani, Toyama, 930-0194 Japan; 2 Department of Urology, Sapporo Medical University School of Medicine, Sapporo, Japan; 3 Department of Urology, Hakodate Goryoukaku Hospital, Hakodate, Japan; 4 Department of Urology, Sunagawa City Medical Center, Sunagawa, Japan; 5 Department of Urology, Tonami general hospital, Tonami, Japan; 6 Department of Urology, Takaoka city hospital, Takaoka, Japan; 7 Department of Urology, National Hospital Organization Kyushu Cancer Center, Fukuoka, Hakata Japan

**Keywords:** Metastatic renal cell carcinoma, Nivolumab, Neutrophil-lymphocyte ratio

## Abstract

**Background:**

The neutrophil-lymphocyte ratio (NLR) is a well-known prognostic marker in various cancers. However, its role as a predictive marker for the effectiveness of nivolumab in patients with metastatic RCC (mRCC) remains unclear. We evaluated the relationships between the NLR and progression-free survival (PFS) or overall survival (OS) in mRCC patients treated with nivolumab.

**Methods:**

The data of 52 mRCC patients who received nivolumab therapy were collected from seven institutes and evaluated. The median follow-up period from treatment with nivolumab was 25.2 months (IQR 15.5–33.2).

**Results:**

The median duration of nivolumab therapy was 7.1 months (IQR 2.9–24.4). The objective response rate was 25% and the 1- and 2-year PFS rates were 46.2 and 25.2%, respectively. The median NLR values at baseline and 4 weeks were 3.7 (IQR 2.7–5.1) and 3.3 (IQR 2.4–5.7), respectively. In the multivariate analysis, an NLR of ≥3 at 4 weeks was an independent predictor of PFS (*P* = 0.013) and OS (*P* = 0.034). The 1-year PFS of patients with an NLR of < 3 at 4 weeks was better than that of those with an NLR of ≥3 (75% versus 29%, *P* = 0.011). The 1-year OS of patients with an NLR of < 3 at 4 weeks was also better than that of those with an NLR of ≥3 (95% versus 71%, *P* = 0.020).

**Conclusions:**

Although the baseline NLR was not associated with PFS or OS, an NLR of ≥3 at 4 weeks after the initiation of therapy might be a robust predictor of poor PFS and OS in mRCC patients undergoing sequential treatment with nivolumab.

## Background

Over the last decade, the treatment of metastatic renal cell carcinoma (mRCC) has dramatically changed. Vascular endothelial growth factor (VEGF) and mammalian target of rapamycin (mTOR)-targeting agents have superseded cytokine therapies. More recently, immune checkpoint inhibitors (ICIs) have emerged as a new treatment option for mRCC. Nivolumab, a programmed cell death protein-1 (PD-1) antibody, induces restoration of the anticancer T cell mediated immune response by blocking PD-1 [1]. In a global multicenter phase 3 trial (CheckMate 025), nivolumab treatment was associated with longer overall survival (OS) in mRCC patients who were previously treated with tyrosine kinase inhibitors (TKIs), in comparison to everolimus [2]. On the basis of these results, the major guidelines recommend nivolumab as a second-line treatment for mRCC patients [3, 4].

Although ICIs, including nivolumab, have resulted in changes to the treatment strategies for mRCC, there are unmet needs for biomarkers to predict the effectiveness of ICIs. The Memorial Sloan Kettering Cancer Center (MSKCC) risk category and International Metastatic Renal Cell Carcinoma Database Consortium (IMDC) risk category are widely used for predicting the prognosis of patients [5, 6]. However, the MSKCC and IMDC risk categories were developed using data from patients who received cytokine therapy and targeted therapy, respectively. Thus, novel prognostic biomarkers or models are required in the ICI era.

Many studies have reported the role of the systemic inflammation response, including C-reactive protein (CRP), albumin, neutrophil, and related variables, for predicting the prognosis of patients with various cancers [7–9], including mRCC [10]. Recently, the neutrophil-lymphocyte ratio (NLR) has been recognized as a predictive marker for both non-metastatic RCC [11–13] and for mRCC treatment with TKI [14–17]. Although some studies [18, 19] revealed that a lower NLR was a significant predictor of favorable progression-free survival (PFS) and OS in mRCC patients treated with ICIs, these studies analyzed data from various lines and ICIs. Thus, there are no robust data about the NLR as a prognostic factor from studies focused on sequential treatment with nivolumab. The aim of this study was to evaluate whether the NLR is a predictor of poor oncological outcomes in mRCC patients undergoing sequential treatment with nivolumab.

## Methods

### Patients’ characteristics (Table 1)

Data from 52 patients who underwent nivolumab treatment between January 2016 and November 2018 were collected from 7 institutes (Sapporo Medical University Urologic Oncology Consortium and Toyama Urologic Study Group), retrospectively. All patients received one or more prior anti-VEGF therapy. The median follow-up period from the initiation of nivolumab treatment was 25.2 months (IQR: 15.5–33.2). The patients consisted of 36 (69%) men and 16 (31%) women (median age, 67.0 years [IQR, 60.2–71.0]).

**Table 1:** Patient characteristics (*n* = 52)

Characteristic		N (%)
Sex	Male	36 (69)
	Female	16 (31)
Prior nephrectomy	Yes	45 (87)
	No	7 (13)
Age at start of nivolumab	Median (range)	67.0 (38–86)
ECOG PS	0	35 (67)
	1	15 (29)
	2	2 (4)
Histologic type	Clear cell carcinoma	42 (81)
	Non-clear cell carcinoma	6 (11)
	Unknown	4 (8)
Treatment line of nivolumab	Second	18 (35)
	Third line or later	34 (65)
IMDC risk classification	Favorable	8 (15)
	Intermediate	37 (71)
	Poor	7 (14)
Number of metastatic sites	< 3	31 (60)
	≥3	21 (40)
CRP at baseline	< 10 mg/L	29 (56)
	≥10 mg/L	23 (44)
NLR at baseline	< 3	20 (39)
	≥3	32 (61)
NLR at 4 weeks	< 3	20 (39)
	≥3	31 (59)
	unknown	1 (2)

*ECOG* Eastern Cooperative Oncology Group; *PS* Performance status; *IMDC* International Metastatic Renal Cell Carcinoma Database Consortium; *CRP* C-reactive protein; *NLR* Neutrophil-to-lymphocyte ratio

Clinical data collected from medical records included demographic information, IMDC risk factors [6], Eastern Cooperative Oncology Group performance status (ECOG PS), treatment line of nivolumab, number of metastatic sites, C-reactive protein at baseline, NLR at baseline and NLR at 4 weeks after the initiation of therapy. Hematological analyses were performed, and the serum chemistry was examined at baseline and at every nivolumab treatment. Response or progression were determined according to the Response Evaluation Criteria in Solid Tumors version 1.1 [20]. The follow-up protocol consisted of computed tomography or magnetic resonance imaging at baseline and every 3 months. This study was approved by the ethics committees of all participating institutions.

### Statistical analyses

OS was calculated from the day of the initiation of nivolumab therapy to the date of death from any cause and was censored at the date of the last follow-up for surviving patients. PFS was calculated from the day of the initiation of nivolumab therapy to the date of documented progression or death (in the absence of progression) and was censored at the last date without any events. The treatment line at the start of nivolumab, number of metastatic organ sites and Eastern Cooperative Oncology Group (ECOG) performance status (PS) were compared using Fisher’s exact test. The IMDC risk classification was compared using the chi-squared test. The OS and PFS were analyzed by a log-rank test and a Cox regression analysis was performed to estimate HRs. The OS and PFS were estimated using the Kaplan-Meier method. The distribution of the NLR values was compared using a paired *t*-test. All statistical analyses were performed using the SPSS software program (version 25.0 SPSS; Chicago, IL, USA). *P* values of < 0.05 were considered to indicate statistical significance.

## Results

The patients’ characteristics are shown in Table 1**.** Of the 52 patients, 18 (35%) and 34 (65%) received one and two or more prior therapeutic treatments, respectively. Eight, 37 and 7 patients had a favorable, intermediate and poor risk IMDC classification, respectively. The median duration of nivolumab therapy was 7.1 months (IQR: 2.9–24.4). The best responses during nivolumab therapy were a complete response in 2 (4%), partial response in 11 (21%), stable disease in 22 (42%) and progressive disease (PD) in 17 (33%) patients. The objective response rate was 25%.

**Fig. 1:**
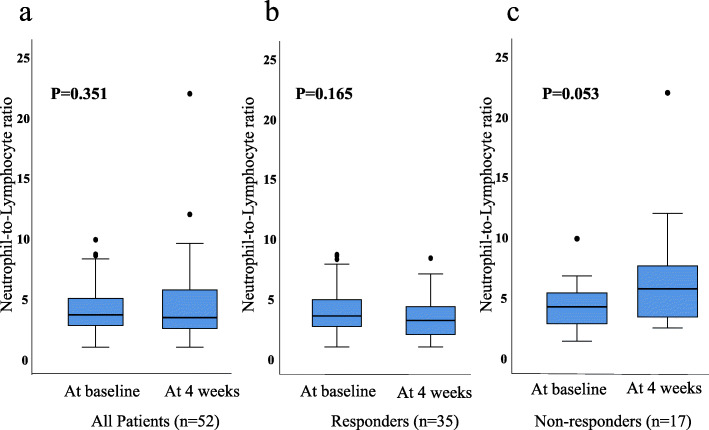
The neutrophil-lymphocyte ratio (NLR) of all patients (**a**), responders whose best response was a complete response (CR), partial response (PR) or stable disease (SD) (**b**) and non-responders whose best response was progressive disease (PD) (c)

The median NLR at baseline was 3.7 (IQR: 2.7–5.1). At baseline, 32 (61%) had an NLR of ≥3 and 20 (39%) patients had an NLR of < 3. The median NLR at 4 weeks after the initiation of nivolumab treatment was 3.3 (IQR: 2.4–5.7). At 4 weeks, 31 (59%) patients had an NLR of ≥3 and 20 (39%) patients had an NLR of < 3. The NLR at 8 weeks after the initiation of nivolumab treatment could not be analyzed in 11 (21%) of the patients because of discontinuation due to progression disease or adverse events. The median NLR at 8 weeks was 3.2 (IQR: 2.3–5.7). The NLR at baseline and that at 4 weeks did not differ to a statistically significant extent (*P* = 0.351). The NLR at 4 weeks and at 8 weeks did not differ to a significant extent (*P* = 0.688). In non-responders (defined as a best response of PD), the NLR at 4 weeks tended to be higher than that at baseline; however, the difference did not reach statistical significance (Fig. 1). Supplementary Table 1 shows predictive factors for patients with NLR of < 3 and NLR of ≥3 at baseline and at 4 weeks after the initiation of nivolumab treatment. There were no significant difference between the proportion of predictive factors and NLR.

### The PFS

**Fig. 2:**
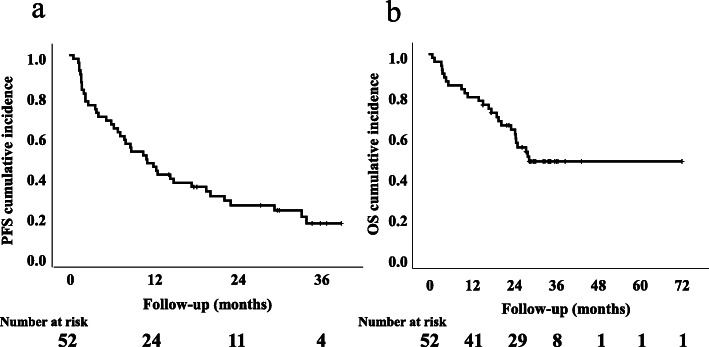
Kaplan-Meier curves for progression-free survival (PFS) (**a**) and overall survival (OS) (**b**) of mRCC patients receiving nivolumab as a sequential therapy

**Fig. 3:**
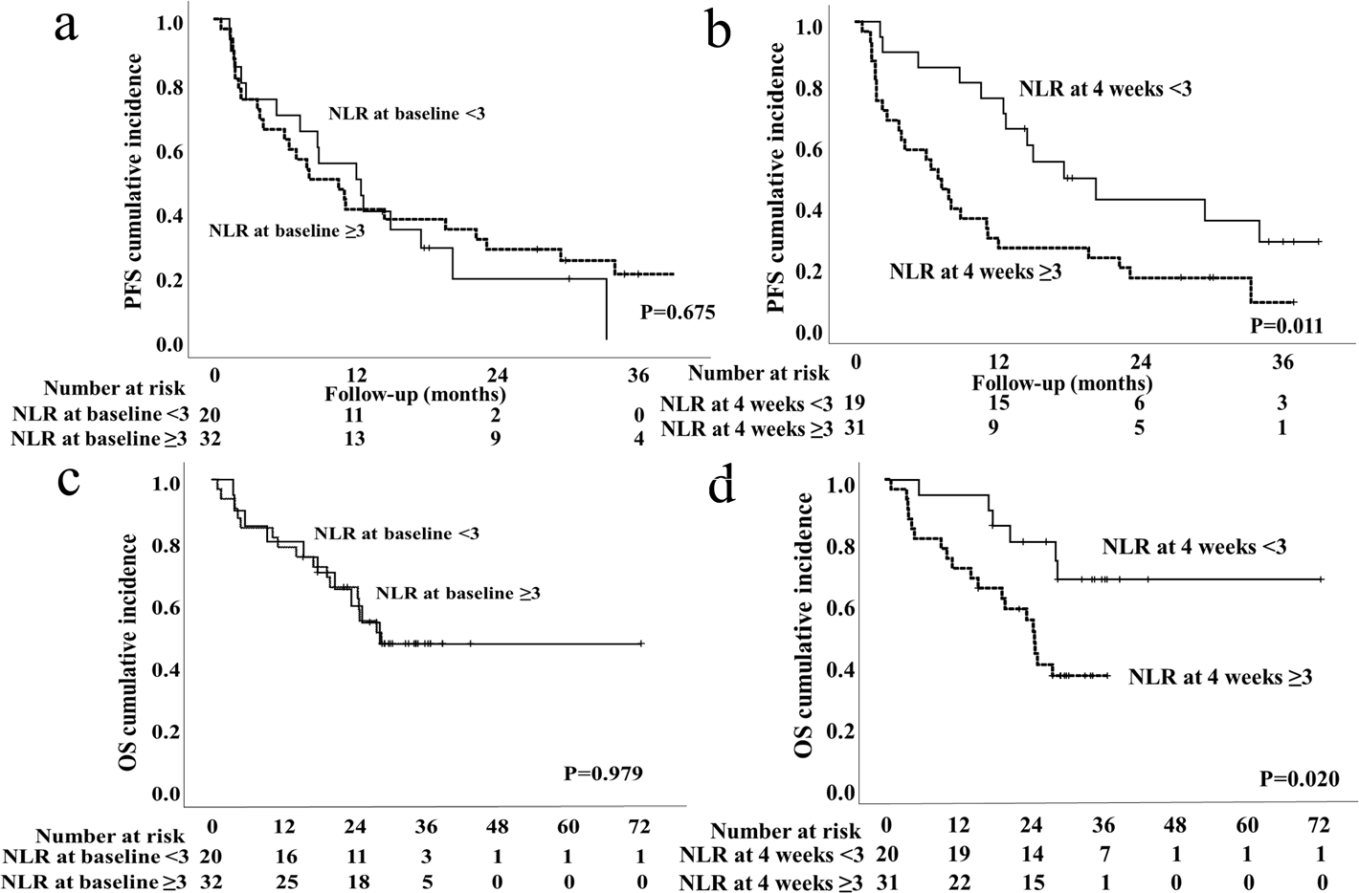
Kaplan-Meier curves for progression-free survival (PFS) of mRCC patients treated with nivolumab stratified by the neutrophil-lymphocyte ratio (NLR) at baseline (**a**), and the NLR at 4 weeks (**b**). Overall survival (OS) stratified by the NLR at baseline (**c**) and that at 4 weeks (**d**)

The 1-year and 2-year PFS were 46.2 and 25.2%, respectively (Fig. 2a). In the univariate analysis of all 52 patients, prior nephrectomy (*P* = 0.046), ECOG PS (0.026), IMDC poor risk (*P* = 0.045), and an NLR ≥3 at 4 weeks (*P* = 0.013) were identified as significant predictors of a poor PFS. In the multivariate analysis, only an NLR ≥3 at 4 weeks (HR 2.340, 95% CI 1.19–4.59, *P* = 0.013) as independently associated with the PFS (Table 2). The 1-year PFS of patients with an NLR of ≥3 and those with an NLR of < 3 at 4 weeks were 29.0 and 75.0%, respectively (*P* = 0.011). A significant difference in PFS was seen between patients with an NLR of ≥3 and those with an NLR of < 3 at 4 weeks, but not at baseline (Fig. 3a and b).

**Table 2:** The results of the univariate analyses and Cox multivariate analyses of factors predicting the PFS (*n* = 52)

	Univariate		Multivariate	
Variables	HR (95% CI)	*p* Value^*^	HR (95% CI)	*p* Value^*^
Age				
(< 70 years vs. ≥70 years)	1.084 (0.57–2.06)	0.804		
Sex				
(Male vs. Female)	1.281 (0.92–1.76)	0.137		
Prior nephrectomy				
(yes vs. no)	2.341 (1.01–5.04)	0.046		
ECOG PS				
(0 vs. ≥1)	2.072 (1.09–3.94)	0.026		
Treatment line of nivolumab				
(2 vs. ≥3)	1.454 (0.77–2.75)	0.251		
IMDC risk classification				
(Favorable, Intermediate vs. Poor)	2.331 (1.02–5.33)	0.045		
Number of Metastatic Organ site				
(1,2 vs. ≥3)	1.023 (0.55–1.92)	0.944		
CRP at baseline				
(< 10 mg/L vs. ≥10 mg/L)	1.001 (0.53–1.89)	0.997		
NLR at baseline				
(< 3 vs. ≥3)	1.147 (0.60–2.12)	0.676		
NLR at 4 weeks				
(< 3 vs. ≥3)	2.340 (1.19–4.59)	0.013	2.340 (1.19–4.59)	0.013

^*^Cox proportional hazards model

*PFS* Progression-free survival; *HR* Hazard ratio; *CI* Confidence interval; *ECOG* Eastern Cooperative Oncology Group; *PS* Performance status; *IMDC* International Metastatic Renal Cell Carcinoma Database Consortium; *CRP* C-reactive protein; *NLR* Neutrophil-to-lymphocyte ratio

### The OS

The 1-year and 3-year OS after nivolumab treatment were 78.8 and 47.2%, respectively. The median OS was 27.9 months (Fig. 2b). In the univariate analysis of all 52 patients, sex female (*P* = 0.004), ECOG PS ≥1 (*P* = 0.021) and NLR ≥3 at 4 weeks (*P* = 0.026) were significant predictors for poor OS (Table 3). In the multivariate analysis, female sex (HR 1.679, 95% CI 1.13–2.50, *P* = 0.011) and an NLR ≥3 at 4 weeks after nivolumab treatment (HR 2.734, 95% CI 1.08–6.92, *P* = 0.034) were independent risk factors for the OS (Table 3). The 1-year OS of patients with an NLR of ≥3 and those with an NLR of < 3 at 4 weeks were 71.0 and 95.0%, respectively (*P* = 0.020). A significant difference was observed in the OS of patients with an NLRs of ≥3 and < 3 at 4 weeks; however, this difference was not observed between patients with these NLRs at baseline (*P* = 0.979) (Fig. 3c and d). The Kaplan-Meier curves of the patients stratified by sex (*P* = 0.002) are shown in Supplementary Fig. 1.

**Table 3:** The results of the univariate analyses and Cox multivariate analyses of factors predicting the OS (*n* = 52)

	Univariate		Multivariate	
Variables	HR (95% CI)	*p* Value^*^	HR (95% CI)	*p* Value^*^
Age				
(< 70 years vs. ≥70 years)	2.307 (0.93–5.76)	0.073		
Sex				
(Male vs. Female)	1.773 (1.20–2.61)	0.004	1.679 (1.13–2.50)	0.011
Prior nephrectomy				
(yes vs. no)	1.190 (0.41–3.46)	0.749		
ECOG PS				
(0 vs. ≥1)	2.481 (1.14–5.38)	0.021		
Treatment line of nivolumab				
(2 vs. ≥3)	1.371 (0.62–3.03)	0.434		
IMDC risk classification				
(Favorable, Intermediate vs. Poor)	1.939 (0.73–5.16)	0.185		
Number of Metastatic Organ site				
(1,2 vs. ≥3)	1.799 (0.83–3.89)	0.135		
CRP at baseline				
(< 10 mg/L vs. ≥10 mg/L)	1.025 (0.47–2.23)	0.951		
NLR at baseline				
(< 3 vs. ≥3)	1.011 (0.46–2.23)	0.979		
NLR at 4 weeks				
(< 3 vs. ≥3)	2.857 (1.14–7.18)	0.026	2.734 (1.08–6.92)	0.034

^*^Cox proportional hazards model

*OS* Overall survival; *HR* Hazard ratio; *CI* confidence interval; *ECOG* Eastern Cooperative Oncology Group; *PS* Performance status; *IMDC* International Metastatic Renal Cell Carcinoma Database Consortium; *CRP* C-reactive protein; *NLR* Neutrophil-to-lymphocyte ratio

## Discussion

This study showed that the NLRs of non-responders at 4 weeks after the initiation of nivolumab tended to be higher than at baseline. We also found that an NLR of ≥3 at 4 weeks was significantly associated with poor PFS and OS. These results suggested that a decline in the NLR may predict a favorable clinical outcome in mRCC patients treated with nivolumab as a sequential therapy.

Cancer-related inflammation is well recognized to be associated with cancer development and a poor prognosis. Tumor-associated neutrophils (TANs) are key regulators of cancer-related inflammation. TANs have been shown to induce genetic instability through the release of reactive oxygen species (ROS) and to produce tumor necrosis factor, IL-1, IL-6 and VEGF, which contribute to tumor proliferation and immune escape [21, 22]. TANs also activate and form neutrophil extracellular traps (NETs), which play a pro-tumor role in tumor progression. TANs are an important factor in the tumor microenvironment, and an increased number of TANs might play a pivotal role in treatment resistance [23].

Boissier et al. performed a meta-analysis to evaluate the prognostic role of the NLR in RCC and demonstrated that a high NLR was associated with a poor prognosis. In patients with both mRCC and localized RCC, an NLR of < 3 predicted better OS and PFS [14]. In non-metastatic clear cell RCC, recurrence-free survival in patients with an NLR of ≥2.7 was significantly shorter than that in patients with an NLR of < 2.7 [11]. Another study on mRCC showed that an NLR of ≥4.0 was an independent predictor of OS [24]. Kobayashi et al. reported that patients with an NLR of < 3.32 had longer PFS in comparison to those with an NLR of ≥3.32 after 1st-line targeted therapy [22].

Several reports have evaluated the role of NLR in the clinical outcomes of mRCC patients were treated with ICI. Jeyakumar et al. showed that an NLR of ≥3 was a predictor of a poor response, PFS and OS in patients with mRCC who were treated with nivolumab, ipilimumab, avelumab, pembrolizumab, or atezolizumab [18]. Lalani et al. reported that a high NLR at baseline and at 6 weeks after the initiation of therapy were associated with short PFS, short OS and a poor response [19]. In our study, the NLR at baseline was not a significant predictor of the response to nivolumab. This may be because all of the patients had received two or more TKI treatments and 63% of them had received three or more such therapies before the initiation of nivolumab treatment. Kobayashi et al. also reported that the NLR significantly decreased, even after treatment with TKIs, especially after sunitinib [22]. However, the NLR before nivolumab treatment was not a significant factor in this study. These results suggested that a decline in the NLR after ICI treatment—rather than TKI treatment—is useful information for predicting a response to nivolumab treatment.

Numerous studies have investigated different cutoff values for the NLR. In mRCC patients, most studies have used a cutoff value of around 3 [15–19, 22, 24–26]. We adopted NLR ≥3 as a significant predictor of poor PFS and OS in patients undergoing sequential treatment with nivolumab, based on the results of the meta-analysis by Hu et al. [15, 18].

The present study is associated with some limitations. First, this was a multi-institutional retrospective study. The patients’ characteristics and treatment strategies might have differed among the institutions. The second limitation was the small number of patients included in this study. Despite these limitations, we believe that these results are clinically informative and will be useful for predicting the clinical outcomes of patients receiving nivolumab as a sequential treatment. However, a prospective study using a larger cohort is needed to confirm this.

## Conclusions

Although the NLR at baseline was not associated with PFS or OS, an NLR of ≥3 at 4 weeks after the initiation of nivolumab therapy could predict poor PFS and OS. However, a larger prospective study is needed to validate these results.

## Supplementary information

**Additional file 1 Supplementary Figure 1.** The Kaplan-Meier curve for the overall survival (OS) of patients stratified by sex.

**Additional file 2 Supplementary Table 1.** Characteristics of patients with an NLR of < 3 or ≥ 3 at baseline and at 4 weeks.

## Data Availability

All datasets on which the conclusions of the manuscript rely are available from the corresponding author on reasonable request.

## Abbreviations

NLR: Neutrophil-lymphocyte ratio
RCC: Renal cell carcinoma
mRCC: Metastatic renal cell carcinoma
PFS: Progression-free survival
OS: Overall survival
ICI: Immune checkpoint inhibitor
PD-1: Programmed cell death protein-1
TKI: Tyrosine kinase inhibitor
VEGF: Vascular endothelial growth factor
MSKCC: Memorial sloan kettering cancer center
IMDC: International metastatic renal cell carcinoma database consortium
ECOG PS: Eastern cooperative oncology group performance status
CRP: C-reactive protein
TANs: Tumor-associated neutrophils
ROS: Reactive oxygen species
NET: Neutrophil extracellular traps
