# Supplementary figures and images for: The neutrophil-lymphocyte ratio has a role in predicting the effectiveness of nivolumab in Japanese patients with metastatic renal cell carcinoma: a multi-institutional retrospective study
Source: BMC Urol. 2020 Jul 25;20:110. doi: 10.1186/s12894-020-00679-2 (PMC7382809; doi:10.1186/s12894-020-00679-2)

## Slide 1
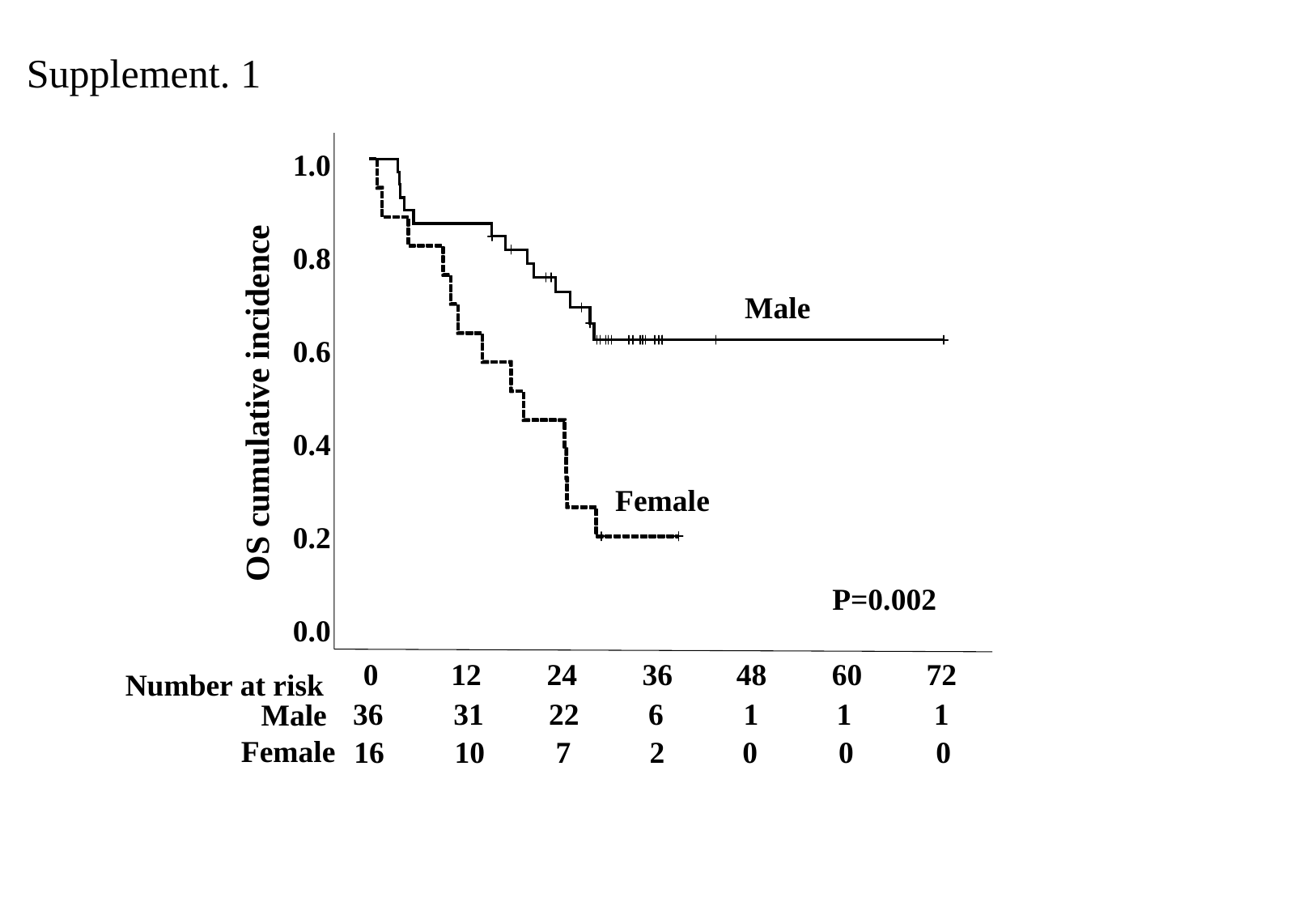

Supplement. 1
1.0
0.8
0.6
0.4
0.2
0.0
0
12
24
36
48
60
72
Male
OS cumulative incidence
Female
P=0.002
Number at risk
36
31
22
6
1
1
1
Male
Female
16
10
7
2
0
0
0

Supplement: Supplementary file 1 — Additional file 1 Supplementary Figure 1. The Kaplan-Meier curve for the overall survival (OS) of patients stratified by sex. [file 12894_2020_679_MOESM1_ESM.pptx]
