# Supplementary material for: The neutrophil-lymphocyte ratio has a role in predicting the effectiveness of nivolumab in Japanese patients with metastatic renal cell carcinoma: a multi-institutional retrospective study
Source: BMC Urol. 2020 Jul 25;20:110. doi: 10.1186/s12894-020-00679-2 (PMC7382809; doi:10.1186/s12894-020-00679-2)
Supplement: Supplementary file 2 — Additional file 2 Supplementary Table 1. Characteristics of patients with an NLR of < 3 or ≥ 3 at baseline and at 4 weeks. [file 12894_2020_679_MOESM2_ESM.docx]

**Supplementary Table 1. Characteristics of patients with an NLR of <3 or ≥3 at baseline and at 4 weeks**

| Characteristic |  | NLR at baseline <3 (n=20) | NLR at baseline ≥3 (n=32) | P value |
| --- | --- | --- | --- | --- |
| Treatment line at start of nivolumab | 2 | 9 (45%) | 9 (28%) | 0.244* |
|  | ≥3 | 11 (55%) | 23 (72%) |  |
| Number of metastatic organ sites | 1,2 | 11 (55%) | 20 (62%) | 0.772* |
|  | ≥3 | 9 (45%) | 12 (38%) |  |
| ECOG PS | 0 | 14 (70%) | 21 (66%) | 1.000* |
|  | ≥1 | 6 (30%) | 11 (34%) |  |
| IMDC risk classification | Favorable | 3 (15%) | 5 (16%) | 0.072** |
|  | Intermediate | 17 (85%) | 20 (62%) |  |
|  | Poor | 0 (0%) | 7 (22%) |  |
|  |  | NLR at 4 weeks <3 (n=20) | NLR at 4 weeks ≥3 (n=31) | P value |
| Treatment line at start of nivolumab | 2 | 5 (25%) | 12 (39%) | 0.373* |
|  | ≥3 | 15 (75%) | 19 (61%) |  |
| Number of metastatic organ sites | 1,2 | 11 (55%) | 19 (61%) | 0.773* |
|  | ≥3 | 9 (45%) | 12 (39%) |  |
| ECOG PS | 0 | 16 (80%) | 12 (39%) | 0.221* |
|  | ≥1 | 4 (20%) | 19 (61%) |  |
| IMDC risk classification | Favorable | 3 (15%) | 5 (16%) | 0.940** |
|  | Intermediate | 15 (75%) | 22 (71%) |  |
|  | Poor | 2 (10%) | 4 (13%) |  |

ECOG, Eastern Cooperative Oncology Group; PS, performance status; IMDC, International Metastatic Renal Cell Carcinoma Database Consortium; NLR, neutrophil-to-lymphocyte ratio.

*: Fischer’s exact test, **: chi-squared test
